# Supporting best practice in reflexive thematic analysis reporting in *Palliative Medicine*: A review of published research and introduction to the *Reflexive Thematic Analysis Reporting Guidelines* (RTARG)

**DOI:** 10.1177/02692163241234800

**Published:** 2024-03-12

**Authors:** Virginia Braun, Victoria Clarke

**Affiliations:** 1 Waipapa Taumata Rau, The University of Auckland, Auckland, New Zealand; 2 University of the West of England, Bristol, UK

**Keywords:** Coding, interpretative, positivism, qualitative, theme, topic summary

## Abstract

**Background:**

Reflexive thematic analysis is widely used in qualitative research published in *Palliative Medicine*, and in the broader field of health research. However, this approach is often not used *well.* Common problems in published reflexive thematic analysis *in general* include assuming thematic analysis is a singular approach, rather than a family of methods, confusing themes and topics, and treating and reporting reflexive thematic analysis as if it is atheoretical.

**Purpose:**

We reviewed 20 papers published in *Palliative Medicine* between 2014 and 2022 that cited Braun and Clarke, identified using the search term ‘thematic analysis’ and the default ‘relevance’ setting on the journal webpage. The aim of the review was to identify common problems and instances of good practice. Problems centred around a lack of methodological coherence, and a lack of reflexive openness, clarity and detail in reporting. We considered contributors to these common problems, including the use of reporting checklists that are not coherent with the values of reflexive thematic analysis. To support qualitative researchers in producing coherent and reflexively open reports of reflexive thematic analysis we have developed the *Reflexive Thematic Analysis Reporting Guidelines* (the RTARG; in Supplemental Materials) informed by this review, other reviews we have done and our values and experience as qualitative researchers. The RTARG is also intended for use by peer reviewers to encourage methodologically coherent reviewing.

**Key learning points:**

Methodological incoherence and a lack of transparency are common problems in reflexive thematic analysis research published in *Palliative Medicine*. Coherence can be facilitated by researchers and reviewers striving to be *knowing* – thoughtful, deliberative, reflexive and theoretically aware – practitioners and appraisers of reflexive thematic analysis and developing an understanding of the diversity within the thematic analysis family of methods.

**What is already known about the topic?**

- Reflexive thematic analysis is widely used to develop themes from qualitative data in health research.
- Braun and Clarke have identified common problems in published thematic analysis research more broadly, including: assuming thematic analysis is one approach, rather than a family of methods; confusing themes and topics; and treating and reporting reflexive thematic analysis as if it is simply a method, without (needing) theoretical foundations that underpin and substantiate the analytic procedures.^1^

**What this paper adds?**

- An interpretative review of 20 papers citing Braun and Clarke published in *Palliative Medicine* between 2014 and 2022.
- The identification of common problems and areas of good practice.
- The grouping of common problems into two domains: (1) a lack of clarity, reflexive openness and detail in reporting; and (2) methodologically incoherent reporting, where research values, methodological practices, quality standards, concepts and language don’t align.
- New *Reflexive Thematic Analysis Reporting Guidelines* (RTARG) – developed in relation to COREQ and SRQR but informed by the values of reflexive thematic analysis – which provide guidance for reporting reflexive thematic analysis with methodological coherence and reflexive openness.
- The recommendation that RTARG be used instead of COREQ or SRQR for reporting reflexive thematic analysis.

**Implications for practice, theory or policy**

- Qualitative researchers need to understand the diversity of theoretical assumptions and procedural practices within the thematic analysis family of methods.
- Qualitative researchers should reflect on their research values and select a thematic analysis approach that coheres with these.
- Qualitative researchers should use a reporting checklist, standards or guidelines that coheres with their thematic analysis approach – RTARG for reflexive thematic analysis.

## Background

Health researchers have access to a plethora of techniques for analysing qualitative data – and thematic analysis has become one of the most widely used of these. The term ‘thematic analysis’ evokes a singular method, but thematic analysis is best conceptualised as a *family* of methods that are focussed on developing and reporting themes from qualitative data. These methods typically involve procedures for coding and theme development, and allow both for inductive analyses, grounded in the data and deductive analyses, guided by existing theory, as well as for coding data for semantic (surface, obvious, explicit) and latent (underlying, assumed, implicit) meaning. Moreover, thematic analysis is commonly understood as closer to a technique, with some degree of theoretical and design flexibility, rather than a theoretically informed and delimited methodology, like most other qualitative analytic approaches (e.g. grounded theory, interpretative phenomenological analysis and narrative analysis). However, it is important to recognise that there are significant divergences in how coding and theme development procedures are conceptualised and enacted, across different versions of thematic analysis and in underlying research values. A distinction we find useful in mapping and making sense of this diversity is Finlay’s demarcation between ‘scientifically descriptive’ and ‘artfully interpretative’ approaches.^2^

*Scientifically descriptive* approaches prioritise scientific research values (based within [post]positivist logics) – evident through practices intended to ensure the reliability or accuracy of coding. Such practices include use of a structured coding process involving a code book or coding frame, developed prior to or early on in the analytic process and the application of this coding frame to the data by multiple coders working independently. The coders use the coding frame to assign data to predetermined ‘themes’. The level of ‘agreement’ between coders is then calculated using statistical tests, with a high level of agreement indicating supposedly reliable coding. Asking participants to validate the analysis as an accurate or faithful representation of their experience is also used to ensure trustworthiness and credibility.

By contrast, *artfully interpretative* approaches (based within non-positivist logics) acknowledge and *embrace* the inherent subjectivity of coding. Quality practice is evidenced through an organic and flexible approach to coding that is responsive to the researcher’s deepening engagement with their data, and their reflexivity. This approach contains the potential for the researcher to question their assumptions and positioning, and how these might be shaping and restricting their understandings, and thus coding can evolve during the process. Themes are developed from and through coding, meaning researchers cannot ‘code for themes’. Participant validation is not coherent with artfully interpretative thematic analysis, because it assumes the possibility of an external reference point from which the ‘accuracy’ of the analysis can be judged.^3^ More appropriate is the concept and practice of member *reflections*,^4^ which create opportunities for elaboration and further insight.

We first developed an artfully interpretative thematic analysis approach in 2006, to provide qualitative researchers with a non-positivist or ‘Big Q’ alternative at a time when most thematic analysis approaches were ‘small q’. A small q/Big Q distinction broadly maps onto Finlay’s scientifically descriptive and artfully interpretative distinction.^5^ Big Q Qualitative involves the use of techniques for generating and analysing qualitative data underpinned and shaped by distinctly qualitative research values or paradigms (e.g. constructivist and interpretative), which embrace researcher subjectivity as a resource for research and view knowledge as situated and partial.^6^ Small q qualitative defines qualitative research as the collection and analysis of qualitative data, typically, if often implicitly, guided by (post)positivism.

With hindsight, we realise we should have more explicitly articulated our goal of providing a distinctly Big Q approach to thematic analysis, and more clearly demarcated our approach from others. This is why we now call our approach *reflexive* thematic analysis. Recognising that our failure to do so no doubt contributed to misunderstandings and misapplications of reflexive thematic analysis, combined with our goal of supporting qualitative researchers in conducting and publishing high quality (reflexive) thematic analysis, led us to deeper reflection on and a refinement and rearticulation of our approach.^6^ In this process, we have interpretatively reviewed ‘samples’ of published research to understand the common problems in published thematic analysis in general^1^ – such as assuming thematic analysis is a singular approach rather than a family of methods, treating and reporting thematic analysis as if it is atheoretical, and confusing themes and topics, and themes and codes – and explored how thematic analysis has been applied in specific areas, like health psychology,^6^ or in specific journals.^7^ We have also developed guidelines to support reviewers and editors, and au-thors, in publishing high quality thematic analysis.^1,8^

## Purpose

To support good practice in reflexive thematic analysis research and reporting in the field of palliative care,^6,9^ we continue this practice. In this paper, we provide an interpretative review of 20 papers citing Braun and Clarke published in *Palliative Medicine*, and present our newly-developed *Reflexive Thematic Analysis Reporting Gui-delines* (RTARG). These guidelines are intended to replace existing checklists – such as COREQ^10^ and SRQR^11^ – which do not support good practice in reporting reflexive thematic analysis. Our aim in the review was to understand and consider the ‘state-of-the-art’ in reflexive thematic analysis reporting in the journal, and to identify patterns of problematic and good practice, from our standpoint as non-positivist or Big Q Qualitative researchers.^5^ We identified papers using the search function on the journal webpage, the search term ‘thematic analysis’, and the default ‘relevance’ setting. We selected the first 20 papers citing any Braun and Clarke thematic analysis source – most were the 2006 paper in which we first outlined our approach,^9^ although a few more recently published papers (also) cited Braun and Clarke,^12^ in which we ex-plained why we now refer to our approach as *reflexive* thematic analysis. We chose the criteria of simply citing Braun and Clarke rather than claiming to ‘follow’ or ‘use’ Braun and Clarke’s approach. Previous reviews^7,13^ have evidenced that it is often very difficult to discern either from citations or the description of the analytic approach, what existing approach, if any, was followed, and also that it is common for authors to claim to ‘follow’ Braun and Clarke, but describe an analytic approach that bears little or no resemblance to reflexive thematic analysis. Because of this blurring, in this paper, we sometimes refer to thematic analysis in general, and sometimes to reflexive thematic analysis specifically. The 20 papers were published between 2014 and 2022. Given our focus is on understanding *common* problems in reporting, to avoid ‘naming and shaming’ individual authors, we do not identify the papers included in the review – though we do identify some specific instances of good practice.

Our interpretative review process involved reading each paper, and making notes on aspects we had previously identified as important to quality reporting of reflexive thematic analysis.^1,13^ These included (but was not limited to): the researcher ‘owning their perspectives’^13^ through detailing their theoretical assumptions and reflexive practice and positionings; the researchers’ account of their analytic process; and the conceptualisation of themes. For some aspects, we simply noted presence/absence of an aspect (and if present, *what* was included). For others, we made a positioned, interpretative judgement about *how* something (e.g. themes, a deductive orientation) was conceptualised (based on the reported content) and/or the quality of reporting. In this way, the review can be understood as a situated, interpretative and subjective, but also rigorous process of engagement.

The development of the RTARG was informed by this and other reviews,^1,13^ a wider scholarly project on quality and Big Q Qualitative reporting,^14,15^ and our experiences and perspectives as reflexive thematic analysis methodologists and qualitative researchers. As our aim is that the RTARG *replaces* the use of checklists like COREQ and SRQR, we reasoned through the individual items/elements in those checklists, considering whether or not they were compatible with reflexive thematic analysis, and thus supported or undermined quality reporting. We retained or reworked items/elements that broadly cohered with reflexive thematic analysis; others, we rejected or replaced. We also read widely on reporting checklists, standards and guidelines and critiques and took from existing guidelines anything useful that wasn’t already captured by our reworking of items from COREQ and SRQR. As authors will tussle with (unknowingly) small q, scientifically descriptive inclined reviewers, we thought it useful to also highlight concepts and practices *to avoid*, the sorts of things we argue are commonly but mistakenly assumed to be generic.

## How thematic analysis was used in the reviewed papers?

The ‘sample’ consisted of 18 empirical studies and 2 systematic reviews. Most of the empirical studies were stand-alone qualitative studies, with one mixed method study and four qualitative studies that were a part of larger mixed method designs/trials. The papers mostly evidenced an experiential orientation to qualitative research – empathically exploring participants’ lived experiences and perspectives or the factors that influence particular phenomena.^6^ Interviews and focus groups were the most common data generation methods (used in 14 papers), but there was some diversity, with researchers harnessing the design flexibility of thematic analysis.^8^ Other methods for data generation included observation, surveys and participatory approaches. The analytic method used was variously described as thematic analysis, thematic content analysis, Braun and Clarke’s thematic analysis and, in more recent papers, reflexive thematic analysis. Thematic analysis was mostly used inductively, even if not labelled as such, with the analysis grounded in the data; there were some examples of a deductive approach where an existing theory or model was used as a framework for/to guide the coding. Our work was typically the sole analytic methodological source cited.^9,12^ In some papers, the analytic procedures described were completely different from those we describe, with phases and procedures added or not engaged with, without any explanation. A few papers cited other sources – for thematic analysis, grounded theory or qualitative content analysis – and these different approaches guided the analysis, sometimes in combination with reflexive thematic analysis. In a few papers, multiple thematic analysis approaches were cited, but it wasn’t obvious that any one approach guided the analysis, or how disparities in philosophy or procedure across the approaches had been addressed.

Indeed, there was no discussion of the theoretical grounding of the use of thematic analysis, and of the research more broadly, in most of the papers reviewed. The theoretical frameworks that were declared or alluded to – interpretative phenomenology/qualitative research, realism and contextualism – were consistent with an experiential orientation to analysis. Examples of explicit discussion of theory included Collins et al.’s^16^ description of their interpretative phenomenological framework: ‘Concerned with the individual participants’ perceptions or account of experiences, an interpretative, phenomenological framework underpinned the design, sampling decisions, interviewing techniques and analysis of interviews’ (p. 951). Pino et al.^17^ described a realist approach: ‘we broadly adopted thematic analysis as a ‘realist’ method, treating what people said as reflecting their views and perspectives. Nevertheless, we also endeavoured to take into account the way in which the interviewer’s questions shaped interviewee responses’ (p. 710). Both examples demonstrate the possibility of succinctly yet cogently describing the theoretical assumptions that underpin an analysis. However, the absence of such description was the norm. Not explicitly discussing theoretical grounding is problematic as thematic analysis does not offer a theoretically informed and delimited methodology. Without a methodological ‘package’ that specifies not just analytic procedures, but also guiding theoretical assumptions, suitable or ideal research questions and data generation techniques and the appropriate constitution of the participant group/dataset, theory needs to be explicitly considered.

Overall, authors tended not to ‘own’ and articulate their perspectives, an important aspect of Big Q research quality.^13^ There was very little discussion of researcher reflexivity in the articles reviewed, other than brief mentions of the professional positioning of the researchers and/or keeping a reflexive diary in a few articles. As a notable exception, Fusi-Schmidhauser et al.^18^ included nice reflections on navigating the power dynamics in their participatory action research:

practising action research within a practitioner’s group with hierarchical relationships is challenging. The risk of developing asymmetrical relationships and thus preventing a truthful capture of all opinions and voices needs to be constantly assessed. The CIG [collaborative inquiry group] presented a dual power imbalance: one between the researcher and the non-medical professionals (nurse, physiotherapist), the second between the researcher, a senior consultant in palliative care and her medical colleagues. Continuous reflexivity and ongoing discussions about power relationships and sources of inequity were helpful to address potential study limitations. In addition, the use of few practicalities, such as participants interacting by first name, creating an informal Smartphone chat-app to schedule all group meetings and attending meetings in plainclothes, thus avoiding white coats within the physician group, helped to overcome potential power imbalance (p. 1938).

None of the reviewed papers were entirely ‘problem’ free – perhaps partly a reflection of constrained word counts and the requirement to use reporting checklists when existing qualitative reporting checklists such as COREQ^10^ and SRQR^11^ tend to exemplify and promote methodological incoherence (see below) – it is notable that more recently published papers, particularly those citing *Reflecting on reflexive thematic analysis*, were often stronger.

In order to make our evaluation of thematic analysis published in *Palliative Medicine* valuable for future authors in a way that facilitates quality, we now highlight the two broad domains that we categorised most problems we identified into:^6,7^ (1) a lack of transparency or reflexive openness,^19^ clarity and detail in reporting; and (2) methodological incoherence in what is reported.^4^ We use the term reflexive openness as an alternative to the more widely used term transparency,^20^ as the concept of transparency ‘rests on an ocular metaphor, implying the possibility of *seeing through* to gain access to things *in themselves* or things *as they really are*’ (p. 181; emphasis in original)^19^ – a metaphor not coherent with a non-positivist approach such as reflexive thematic analysis.

## Lack of reflexive openness, clarity and detail

We only briefly touch on the first domain, which we refer to as lack of (adequate) reflexive openness, clarity and detail in the reporting of the research, such as limited detail about the authors’ analytic process, or inconsistency in the presentation of the themes. For instance, clear maps or tables are a really useful tool for clearly communicating the overall analytic structure (see Jämterud and Sandgren^21^ and Johnson et al.^22^), but if authors use different and/or additional theme names/headings in the developed analysis, confusion is created. There are obvious challenges in reporting qualitative research in journals with tight word limits, such as *Palliative Medicine* with a maximum of 3000 words excluding data extracts, tables and references. Reflexive thematic analysis does not provide a strict method – ‘recipe’ to follow, but requires situated and reflexive researcher engagement. This means researchers (ideally) need to report not the generic phases (which is often what was reported in articles reviewed), but their specific engagement, process and decisions in using the approach – which requires words. Hanna et al.^23^ offered a good *brief* example of this from the reviewed papers (for in-depth examples see *Student Examples of Good Practice* [under *Quality*] on our website www.thematicanalysis.net):

Initially, JRH [the first author] read and reread the transcripts to gain a sense of each professional’s experience. JRH produced written reflections after reading each transcript, outlining thoughts about the individual *story*. Then, JRH manually coded the data, detailing inductive descriptive codes by marking similar phrases or words from the professionals’ narratives. Reflexive thematic analysis was a useful approach to enabling JRH to reflect and engage with the data, generating themes from the codes using mind mapping techniques. The written reflections aided constructing the themes (p. 1251).

In relation to psychology, Levitt et al. argued that reporting qualitative research with integrity requires more space.^24^ They recommended the use of Supplemental Materials to provide more detailed methodological information, including research materials, and comprehensive demographic information, and extended ‘Results’ with further data extracts. Such recommendations offer the scope for shorter-length articles in journals like *Palliative Medicine* to provide greater reflexive openness, clarity and detail, which also (helpfully) decreases the risk of methodological incoherence – the main focus of our discussion here.

## Methodological incoherence

The notion of methodological *coherence* captures research where the different elements – the research question and purpose, the guiding theoretical assumptions (whether explicitly stated or implicitly evident), data generation methods, data analysis method/ologies, quality practices and concepts – are in conceptual alignment.^24^ Incoherence is evident when elements are misaligned (without acknowledgement or discussion). All reviewed articles evidenced some degree of methodological incoherence (often combining together non-positivist and (post) positivist research practices and values, seemingly unknowingly). Here we highlight what we think are key contributors to methodological incoherence in articles reviewed, and offer clarifications as a starting point for future authors to avoid incoherence.

Not realising both the diversity within the thematic analysis family of methods, and the conceptual importance of this, was likely a key contributor to the methodological incoherence evident. The differences do matter, and we argue that it is not possible to coherently combine together scientifically descriptive and artfully interpretive thematic analysis (others disagree^25^) – we offer a fuller discussion of differences in thematic analysis approaches elsewhere.^1,6^ Our recommendation for methodological conference is that qualitative researchers reflect on their research values, choose a thematic analysis approach that aligns with these, and then strive to use, and report, this approach in an aligned and thus coherent way.

A lack of clarity around (the different conceptualisations of) a theme was another source of methodological incoherence in the articles reviewed. Across thematic analysis approaches, we have identified two quite different constructs: (a) topic summaries; and (b) shared-meaning based themes. In scientifically descriptive thematic analysis, themes tend to be conceptualised as summaries of topics. What unites the observations and illustrative data extracts in the ‘theme’ is the topic (e.g. ‘Barriers to. . .’ and ‘Experiences of. . .’), but the observations might capture quite disparate barriers or experiences. (Such themes are developed early in the analytic process – they *can* be developed early precisely because they capture (broad) topic areas or issues; they can be regarded as ‘inputs’ into the analytic process.)

In the articles reviewed, topic summary-type ‘them-es’ were common, and often presented with several sub-themes that each captured a different element of the topic – for example, a different type of barrier. There was rarely an overall story that drew together the different barriers into a coherent thematic statement.^26^ Another – related – conceptualisation of themes typical of scientifically descriptive approaches is of themes as ‘diamonds scattered in the sand’ (p. 740).^27^ Here, themes are often implicitly treated as real things that exist in data and which the researcher ‘identifies’ or ‘discovers’. The language researchers use around theme development can unintentionally signal a conceptualisation of themes as ‘diamonds’, and position their role in theme development as a relatively passive one of ‘finding’ (they can even write themselves out of the process entirely by noting only that ‘themes emerged’).

In artfully interpretative thematic analysis, themes are conceptualised as patterns of shared meaning, organised around a central concept or idea. What unites the observations within a theme is this shared idea or meaning from the dataset (see the examples below), rather than a shared topic. Such themes can be regarded as the endpoints and outputs of the analytic process. As previously noted, rather than guiding coding, they are developed from and through coding, crafted by researchers actively developing and generating themes through labour-intensive open analytic processes. Many of the reviewed articles (incohently) used reflexive thematic analysis to develop and report topics, categories or domains, rather than shared-meaning based themes. For methodological coherence, we think researchers wanting to report topic summaries are better off using an approach that has the development and reporting of topics as its explicit purpose – and where the procedures are oriented to that purpose (e.g. such as framework analysis^28^). To us, the labour-intensive procedures of reflexive thematic analysis make little sense when the ‘thematic structure’ could have been developed early on (as in scientifically descriptive thematic analysis).

Some articles did use reflexive thematic analysis coherently with regard to the conceptualisation and reporting of themes. For example, Collins et al.^16^ reported four themes capturing parents’ experiences of caring for a child with a life-limiting condition. Their evocatively titled themes were: (1) trapped inside the house (which captured parents’physical and social isolation from the community and exclusion from the workforce, and the negative impacts of this on their well-being); (2) the protector (which captured the enormity and responsibility of the caring role); (3) living with the shadow (which described the pervasiveness of living with the probability of their child’s death and grief for the life that could have been); and (4) travelling a different pathway (which described the way the parents derived meaning and purpose from their carer role). Jämterud and Sandgren, who sought to understand the factors that influenced healthcare professionals’ decisions about identifying patients for serious illness conversations, reported four themes: (1) the right patient (the main characteristic of which was physical deterioration); (2) the right time (when the patient knows they are going to die); (3) continuity in relations and continuity over time (an established relationship is necessary both to identify patients and to have the conversation); and (4) death and its relation to hope (serious illness conversations risk taking away hope).^21^ These examples take us beyond topics, and even just the theme *names* give a sense that there is a story being told. They also nicely illustrate two different types of research question reflexive thematic analysis can be used to address – lived experience and influencing factors.^6^

Although most of the reviewed articles did not include any overt/deliberative discussion of theory, theory offered another more subtle form of incoherence – specifically, through (disciplinary dominant) (post) positivist assumptions expressed in language and quality criteria. We have elsewhere referred to such likely undeliberate, unknowing entanglements with (post) positivism as ‘positivism creep’.^6^ We argue that such creep is quite common, even in artfully interpretative thematic analysis – because (post) positivism has inflected (or infected) many of our ideas about (assumed generic) *good* practice. In the reviewed articles, for example, authors made reference to concepts, concerns and practices that reflect (post) positivist concerns – and ones which are often entirely normalised within disciplinary contexts.^29,30^ For example, some described ‘small samples’ or a lack of (statistical) generalisability as limitations of the research; some described saturation as a criterion for stopping data generation (unsurprisingly perhaps, as saturation is widely advocated for and referenced in both COREQ and SRQR; see Braun and Clarke,^31^ for a critique where reflexive thematic analysis is concerned); some reported using quality practices like triangulation of researchers or data sources, and/or participant validation of ‘findings’; some expressed concern about researcher ‘bias’, and reported measures taken to manage researcher bias and ensure the reliability and accuracy of the analysis. As concepts and practices that are founded in (post) positivism, these are incoherent with reflexive thematic analysis.^1^ Qualitative scholars like Varpio et al.^29,30^ have pointed out that the entanglements with (post) positivism that gave qualitative research legitimacy within medicine in its infancy are now hampering the methodological integrity of non-positivist Qualitative research.

Some reviewed papers did evidence coherent quality practices. Mayland et al.,^32^ for example, described their reflexive and collaborative, rather than consensus, app-roach to theme development – noting how the two authors who led the analysis ‘collaboratively reviewed and revised the themes, in conjunction with the quantitative data, and with the wider research team through critical dialogue’ (p. 1482). And Olsman et al.^33^ reflected on the inclusion of ‘studies with different methods and epistemological foundations’ in their interpretative re-view, and referenced the qualitative concept of transferability: ‘in our view, this multiplicity may reinforce the transferability of the findings, although there are different opinions on combining results of studies with different methods’ (p. 67).

Our view is that the problem of methodological incoherence in (reflexive) thematic analysis reporting is not helped by the use of reporting checklists like COREQ^10^ and SRQR,^11^ which just over a third of the articles cited. Although these are presented as generic, they are not well aligned with Big Q Qualitative or artfully interpretative/reflexive thematic analysis.^14,15^ Further, they demonstrate methodological incoherence. COREQ, for example, references both the small q/(post) positivist concept of ‘researcher bias’ (implying a distortion of ideally objective knowledge) and the Big Q/non-positivist concept of ‘reflexivity’ (where knowledge generation is understood as inherently subjective and the researcher inescapably shapes the knowledge produced, they are part of the research rather than separate from it) – without acknowledging or discussing the different values informing these concepts. If an unknowing researcher follows these checklists, they risk methodological incoherence through adherence to practices with quite divergent foundations.

## Supporting better practice in reporting reflexive thematic analysis: Introducing our new *Reflexive Thematic Analysis Reporting Guidelines* (RTARG)

As qualitative methodologists, our aim is to support knowledge, understanding and (consequently) high quality practice – and reporting – of Big Q research. We have deployed the idea of an ‘unknowing’ researcher in this paper, to highlight that we are all situated within different knowledge norms, contexts and experiences, at different places on a qualitative journey. Whether our journey with thematic analysis involves small q or Big Q approaches, we hope to encourage and support researchers to aspire for knowingness (as reflexive openness). Focussing now on Big Q approaches, we end this paper by introducing a new tool to guide the *methodologically coherent* reporting of reflexive thematic analysis.^1,7,34^ This tool – which we are calling *Reflexive Thematic Analysis Reporting Guidelines* (RTARG; see Supplemental Materials) – offers an extension of guidance that we have developed for quality in reflexive thematic analysis, and for Big Q Qualitative more widely.^1,15^ We propose that the RTARG replaces existing widely-used checklists like COREQ^10^ and SRQR,^11^ for reporting reflexive thematic analysis. Intended for use primarily by authors, the guidelines also offer a tool for methodologically coherent *reviewing*. Evaluation of quality and reporting expectations must be grounded in recognition of the diversity within qualitative research and that ‘one size does not fit all’.

Both COREQ and SRQR were developed through a synthesis of existing quality and reporting standards and checklists and/or ‘expert consensus’ – an approach we believe is a problematic basis for determining reporting and quality standards in qualitative research, because of the diversity of practice.^14,15^ Our position is that developing checklists through synthesis and consensus can result in quality and reporting standards that unknowingly invite and encourage methodological incoherence. Our guidance for reporting reflexive thematic analysis is *values based*. For us, aligning with a notion of methodological coherence or integrity as a key principle for quality, values should be the foundation for quality and reporting guidelines.^24^ The RTARG is intended to facilitate reporting that is methodologically coherent with the Big Q/artfully interpretive values of reflexive thematic analysis. RTARG offers *guidelines* and *not* a checklist, as not every item will be relevant to a particular study/report – and there needs to be flexibility around the use of the RTARG.^35^ Unthinking compliance with the RTARG will not necessarily improve reporting quality, because *knowing* practice is key.^7^ We hope that for health journals, where the use of reporting standards and checklists is common, the RTARG can also be used for conceptually and methodologically coherent reviewing of reflexive thematic analysis articles.

## Conclusion

In reviewing published papers that cite Braun and Clarke and report ‘thematic analysis’ – both in *Palliative Medicine* and elsewhere – we have identified common (repeated) problems in reporting – both around conceptualisation and reported practice. We theorised the methodological incoherence as resulting from a range of sources, including the assessment tools (such as checklists like COREQ) and disciplinary norms and expectations that form not only scholars, but the community of reviewers (and editors) – which we can understand as collective forces shaping individual papers. We aim to support all involved in the publication process and facilitate better quality thematic analysis in *Palliative Medicine* and more broadly. To do so, we have provided some brief examples of good practice, highlighted the importance of reflexive openness and methodological coherence, and developed a new tool – the RTARG – for guiding quality practice in reporting reflexive thematic analysis. We know this tool is aspirational – that many publishing limits (such as wordcounts) work against best practice. Yet we remain optimistic for change – not least because the invitation to submit this review, and the publication of the RTARG, suggests an eagerness to understand, explore and facilitate the publication of quality thematic analysis across its diversity.

## Supplemental Material

sj-pdf-1-pmj-10.1177_02692163241234800 – Supplemental material for Supporting best practice in reflexive thematic analysis reporting in Palliative Medicine: A review of published research and introduction to the Reflexive Thematic Analysis Reporting Guidelines (RTARG) Supplemental material, sj-pdf-1-pmj-10.1177_02692163241234800 for Supporting best practice in reflexive thematic analysis reporting in Palliative Medicine: A review of published research and introduction to the Reflexive Thematic Analysis Reporting Guidelines (RTARG) by Virginia Braun and Victoria Clarke in Palliative Medicine
